# Evaluation of Screw Loosening in Patients Undergoing Semi-rigid Stabilization with Polyetheretherketone (PEEK) Rods

**DOI:** 10.1007/s43465-026-01734-0

**Published:** 2026-03-06

**Authors:** Nimetullah Alper Durmus, Ali Sahin, Sukru Oral, Halil Ulutabanca, Ahmet Kucuk, Rahmi Kemal Koc

**Affiliations:** 1 Department of Neurosurgery, Erciyes University Faculty of Medicine, 38280 Talas, Kayseri, Turkey; 2 Department of Neurosurgery, Private Erciyes Hospital, Kayseri, Turkey

**Keywords:** Dynamic stabilization, Lumbar degenerative disease, PEEK rod, Semi-rigid stabilization, Screw loosening

## Abstract

**Background Context:**

Polyetheretherketone (PEEK) rods, designed for semi-rigid stabilization, offer a lower elastic modulus than metallic rods, promoting physiological load sharing and reducing hardware-related complications. Nevertheless, their clinical efficacy—particularly regarding pedicle screw loosening—remains inadequately studied.

**Purpose:**

The aim of this study was to determine the rate of screw loosening and to evaluate potential contributing factors in patients who underwent semi-rigid stabilization with PEEK rods for lumbar degenerative pathologies.

**Study Design/Setting:**

A retrospective cohort study.

**Methods:**

The data of 58 patients who underwent semi-rigid stabilization using PEEK rods between 2017 and 2023 and had a minimum follow-up of 12 months were retrospectively analyzed. Demographic characteristics, surgical details, preoperative and postoperative pain scores, and bone mineral density were measured using dual-energy X-ray absorptiometry (DXA). Screw loosening was assessed based on plain radiographs and computed tomography (CT) imaging.

**Results:**

The study population consisted of 67.2% female patients, with a mean age of 60.4 ± 12.7 years. The screw loosening rate was 13.8%, with six cases attributed to deficiency in preoperative planning, one case to Parkinson’s disease-related physical rigidity, and one case with an osteoporosis. A significant association was found between screw loosening and lower DEXA values as well as higher postoperative VAS scores (*p* < 0.001). No significant differences were observed between screw loosening and diagnosis, gender, or surgical segment length.

**Conclusions:**

Semi-rigid stabilization using PEEK rod systems maintains screw loosening rates within acceptable limits. However, surgical technique errors and low bone quality significantly increase the risk of loosening. Careful patient selection is critical for minimizing complication rates. PEEK rods should not be applied to patients with severe instability, anterior column insufficiency, and osteoporosis.

## Introduction

Degenerative disorders of the lumbar spine, which become increasingly prevalent with advancing age, pose substantial clinical challenges by significantly impairing both quality of life and daily functional capacity [1, 2]. Among the most frequently encountered conditions are lumbar spinal stenosis, degenerative spinal deformities, and recurrent lumbar disc herniations [3, 4]. In advanced stages where conservative management proves insufficient, surgical decompression and stabilization are often necessitated. Although traditional posterior instrumentation techniques employing titanium rods achieve robust fixation, they are associated with long-term complications, including screw loosening, rod fracture, and adjacent segment degeneration [5, 6].

The semi-rigid stabilization systems fabricated from polyetheretherketone (PEEK) have been introduced to mitigate these complications and to better replicate physiological load distribution [7, 8]. Owing to their lower elastic modulus compared to metallic constructs, PEEK rods are designed to reduce stress shielding, enhance biomechanical integration at the screw–bone interface, and thereby diminish the risk of screw loosening [6, 9]. Moreover, it has been hypothesized that semi-rigid systems may attenuate stress transmission to adjacent spinal segments, potentially delaying the progression of degenerative changes [10, 11].

Nevertheless, the efficacy of PEEK rod systems in preventing implant-related complications, particularly screw loosening, remains a subject of ongoing debate within the literature. While some studies suggest that PEEK constructs may confer a protective effect against screw loosening, others argue that this advantage may be attenuated in patients with diminished bone mineral density, wherein micromotion at the implant interface could still precipitate loosening over time [12–15].

The study aimed to analyze demographic and clinical variables, surgical characteristics, and postoperative complications, with particular emphasis on identifying potential risk factors for screw loosening.

## Materials and Methods

This study was conducted through a retrospective review of patients who underwent surgical treatment with semi-rigid stabilization using PEEK rod systems (Osimplant Spine Restoration Technology, Ankara) for lumbar degenerative pathologies between 2017 and 2023. Patients who had completed at least 12 months of postoperative follow-up and for whom complete preoperative and postoperative clinical and radiological records were available were included. Cases with a history of vertebral fracture, spinal tumor, infection, or prior spinal instrumentation were excluded. All surgical procedures were performed by the same surgical team, utilizing a standard posterior approach and a single type of PEEK rod system.

The surgical indications were determined based on the presence of lumbar spinal stenosis, degenerative deformity, recurrent disc herniation, instability or adjacent segment disease causing neurological symptoms or functional limitations unresponsive to conservative treatments. During surgery, decompression with hemilaminectomy of the affected segment was followed by transpedicular screw placement and stabilization with PEEK rods. The length of the stabilized segment was determined according to the extent of pathology, the degree of deformity, and the level of segmental instability. All patients were mobilized after 8 h after surgery. The rest was recommended during 3 weeks. Lumbar sacral corset was recommended for 2-months.

**Fig. 1:**
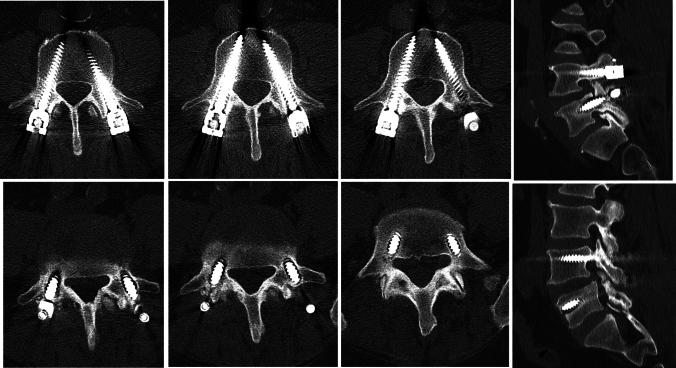
Postoperative (PO) 1.5 years: recurrent low back pain for the past 6 months. Loosening of L5 pedicle screws detected

Demographic data, clinical complaints, radiological findings, and surgical characteristics of the included patients were systematically recorded. Preoperative and postoperative levels of back pain were assessed using the Visual Analog Scale (VAS). Bone mineral density was measured preoperatively by dual-energy X-ray absorptiometry (DXA). Screw loosening was defined radiologically as the presence of a circumferential radiolucent zone measuring ≥ 1 mm around the pedicle screw on plain radiographs and/or computed tomography (CT) scans. CT imaging was used to confirm suspected cases or equivocal findings on plain radiographs (Fig. 1).

All data were recorded and analyzed using SPSS version 22.0 (IBM Corp. Armonk, NY, USA). Descriptive statistics included frequency, percentage, mean, standard deviation, median, minimum, and maximum values. For categorical variable comparisons, Fisher’s exact test was applied when expected frequencies were less than five. In cases where the data did not follow a normal distribution, the Mann–Whitney *U* test was used for independent group comparisons. A *p* value of < 0.05 was considered statistically significant for all analyses.

This research was approved by the local ethics committee and conducted in accordance with the principles of the Declaration of Helsinki.

## Results

Of the 58 patients included in the study, 39 (67.2%) were female and 19 (32.8%) were male. The mean age was 60.4 ± 12.7 years (range 30–86 years). At presentation, 91.4% (*n* = 53) of the patients reported low back pain. The most frequent diagnosis was lumbar spinal stenosis (34.5%), followed by lumbar degenerative deformity (31.0%), recurrent lumbar disc herniation (LDH) (22.4%), and adjacent segment disease (12.1%) (Table 1). There was no statistically significant difference in gender distribution among the diagnostic groups (*p* > 0.05). However, lumbar degenerative deformity and lumbar stenosis were significantly more common in patients aged 60 years and older (*p* < 0.05). No significant differences were observed between diagnostic groups regarding preoperative low back pain (*p* > 0.05) (Table 1).

**Table 1:** Diagnosis of patients with respect to age, gender, and preoperative complaints

Variables	All patients	LSS (*n* = 20)	LDD (*n* = 18)	Re LDH (*n* = 13)	ASD (*n* = 7)	p*
Female	39 (67.2%)	14 (70.0%)	14 (77.8%)	7 (53.8%)	4 (57.1%)	0.498
Male	19 (32.8%)	6 (30.0%)	4 (22.2%)	6 (46.2%)	3 (42.9%)	
Age < 60	24 (41.4%)	7 (35.0%)	4 (22.2%)	9 (69.2%)	4 (57.1%)	0.047
Age ≥ 60	34 (58.6%)	13 (65.0%)	14 (77.8%)	4 (30.8%)	3 (42.9%)	
Preop LBP	53 (91.4%)	18 (90.0%)	18 (100.0%)	10 (76.9%)	7 (100.0%)	0.126
Preop LP	55 (94.8%)	19 (95.0%)	16 (88.9%)	13 (100.0%)	7 (100.0%)	0.737

*LSS* lumbar stenosis, *LDD* lumbar degenerative deformity, *Re LDH* recurrent lumbar herniated nucleus pulposus (rLDH), *ASD* adjacent segment disease, *LBP* low back pain, *LP* leg pain

^*^Fisher’s exact test was used; column percentages are presented

**Fig. 2:**
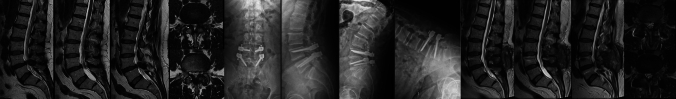
Left-sided L4–5 hemilaminectomy with bilateral decompression and instrumentation using semi-rigid stabilization (PEEK rods)

Postoperative low back pain was present in 47 patients (81%), and all patients with lumbar degenerative deformity underwent long-segment surgery, whereas patients with recurrent LDH and adjacent segment disease more commonly underwent short-segment stabilization (Fig. 2), and this difference was statistically significant (*p* < 0.001) (Table 2). Although screw loosening and postoperative recurrent LDH were more frequent among patients with lumbar degenerative deformity, the difference was not statistically significant (*p* > 0.05). Postoperative low back pain was least frequent in the recurrent LDH group (*p* < 0.05). (Table 2).

**Table 2:** Surgical and postoperative complication status by diagnosis

Variables	All patients	LSS (*n* = 20)	LDD (*n* = 18)	Re LDH (*n* = 13)	ASD (*n* = 7)	p*
Short segment	22 (37.9%)	10 (50.0%)	0 (0.0%)	8 (61.5%)	4 (57.1%)	< 0.001
Long segment	36 (62.1%)	10 (50.0%)	18 (100.0%)	5 (38.5%)	3 (42.9%)	
Screw loosening	8 (13.8%)	2 (10.0%)	5 (27.8%)	1 (7.7%)	0 (0.0%)	0.300
Postop LBP	47 (81.0%)	17 (85.0%)	17 (94.4%)	7 (53.8%)	6 (85.7%)	0.043

*LSS* lumbar stenosis, *LDD* lumbar degenerative deformity, *Re LDH* recurrent lumbar disc herniation (rLDH), *ASD* adjacent segment disease, *LBP* low back pain

^*^Fisher’s exact test was used; column percentages were calculated

**Fig. 3:**
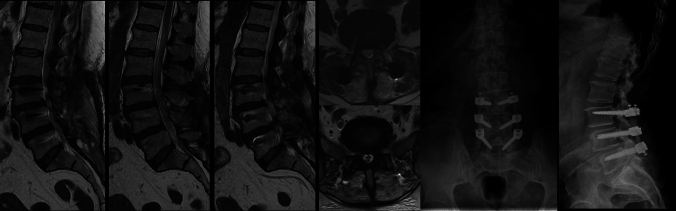
L3–4 and L4–5 hemilaminectomy with bilateral decompression; L3–L5 instrumentation with semi-rigid stabilization using PEEK rods. No complaints at postoperative (PO) 3rd month follow-up

Long-segment (more than two vertebral segments) surgery was performed in 36 patients (62.1%) (Fig. 3). Screw loosening was identified in eight patients (13.8%). When the causes of screw loosening were examined, one patient had flat low back, one patient had intolerable scoliosis, one patient had laminectomy, one patient was elderly (78 years) with anterior column insufficiency, two patients had anterior column insufficiency, one patients was elderly (72 years) with osteoporosis, and one patient was elderly (78 years) with Parkinson’s disease-related physical rigidity. In four of these, the screws were removed, in two the number of screws was reduced. In two, the PEEK rods were replaced with titanium screws, and fusion surgery was performed. All of these patients experienced clinical relief after instrument removal or revision. Revision procedures were indicated in patients with screw loosening who presented with persistent or worsening low back pain and/or radiological signs of mechanical instability despite conservative management; no revisions were performed solely based on radiological findings, and none were required due to neurological deterioration.

When surgical techniques were compared across groups, no significant differences were observed regarding gender, age groups, postoperative screw loosening, postoperative disc herniation, or postoperative low back pain (*p* > 0.05).

The mean time to detection of screw loosening was 11.0 ± 1.5 months (range 8–13 months), while the mean DXA score was − 1.42 ± 0.67 (range − 2.40 to 1.00).

Among patients with screw loosening, there were no significant differences in preoperative low back pain VAS scores compared to patients without loosening (*p* > 0.05). However, late postoperative low back pain VAS scores were significantly higher in patients with screw loosening (*p* < 0.001). In addition, DXA values were significantly lower in the screw loosening group, while there was no significant difference in age between the two groups (*p* > 0.05) (Table 3).

**Table 3:** Screw loosening and various variables

Variables	Screw loosening present Median (min–max)	Screw loosening absent Median (min–max)	*p* value
Preoperative back pain (VAS score)	8 (7–8.1)	8 (7–8.3)	0.938
Postoperative back pain (VAS score)	6 (5–7)	2 (1.6–5)	< 0.001
DEXA	− 1.8 (− 2.3/− 1.5)	− 1.4 (− 2.4/1.0)	< 0.001
Age	64.5 (48–78)	61.5 (30–86)	0.550

Mann–Whitney *U* test was used for statistical comparison

## Discussion

Pedicle screw and rod systems employed for posterior stabilization are critical determinants of surgical success in the management of degenerative lumbar spine disorders [16–20]. In recent years, PEEK based semi-rigid stabilization systems have been proposed as alternatives to conventional titanium rod systems, with the potential to reduce complications such as screw loosening and adjacent segment degeneration [21–24]. In this retrospective study evaluating 58 patients who underwent semi-rigid stabilization using PEEK rods, the overall rate of screw loosening was found to be 13.8%.

The screw loosening rate observed in our study (13.8%) is consistent with previously reported series involving PEEK rod systems [25, 26]. In a 4-year follow-up study conducted by Ohba et al., a screw loosening rate of 15.2% was reported [27]. That study noted that the majority of loosenings were detected within 12 months postoperatively, with some screws demonstrating spontaneous restabilization over time. Similarly, Jıang et al. observed early radiolucent zones around screws in 15.8% of patients treated with PEEK rods, although most cases did not progress to clinically significant instability [25]. Our findings also indicate that screw loosening predominantly occurred within 1 year after surgery and did not show progressive deterioration during long-term follow-up. Nevertheless, the contribution of technical errors during surgical construction to screw loosening was clearly evident. Although semi-rigid stabilization with PEEK rods has been proposed to mitigate long-term adjacent segment disease, such effects are typically observed over extended follow-up periods. In contrast, pedicle screw loosening represents an early implant-related complication, most frequently occurring within the first postoperative year. Therefore, the follow-up duration in the present study is adequate for evaluating screw loosening but insufficient for drawing conclusions regarding long-term adjacent segment pathology.

In our study, when the causes of screw loosening are evaluated, it is observed that there is a deficiency in preoperative planning in all of them such as flat low back, intolerable scoliosis, laminectomy, advanced age, anterior column insufficiency, osteoporosis and Parkinson’s disease-related physical rigidity. The choice of PEEK rod-based semi-rigid stabilization in these cases was made based on the assumption of limited instability at the time of preoperative evaluation. However, subsequent clinical and radiological follow-up demonstrated that sagittal imbalance or anterior column insufficiency had been underestimated in certain patients, resulting in biomechanical conditions exceeding the capacity of semi-rigid constructs. These findings underscore that screw loosening in such cases reflects suboptimal preoperative planning rather than an inherent limitation of the PEEK rod system itself. Accordingly, PEEK rods should be reserved for carefully selected patients with preserved sagittal alignment, adequate anterior column support, and limited instability. Screw loosening can be reduced with good preoperative planning. PEEK rods should not be applied to patients with severe instability, anterior column insufficiency, and osteoporosis. It can be used more safely in limited instability.

In our study, patients diagnosed with lumbar degenerative deformity underwent long-segment stabilization more frequently than other diagnostic groups. However, there was no statistically significant difference in screw loosening rates among different diagnostic categories (*p* > 0.05). While the literature suggests that long-segment fixation increases mechanical stress on screws, thereby elevating the risk of loosening, the lower elastic modulus of PEEK rods appears to mitigate this risk to some extent [12, 28]. Supporting this, Li et al. demonstrated that the use of PEEK rods in long-segment constructs reduced stress shielding and yielded lower screw loosening rates compared to titanium systems; however, this finding contradicts our results [6]. Although a level-specific statistical analysis was not feasible due to the limited number of loosening cases, loosened screws were more frequently observed at junctional levels exposed to higher mechanical stress, such as the lower instrumented vertebrae. This observation suggests that screw location may play a role in loosening risk and warrants further investigation in larger, prospective studies.

Assessment of bone mineral density revealed that patients with screw loosening had significantly lower DXA scores (*p* < 0.001), highlighting low bone quality (osteopenia) as a significant risk factor for screw loosening. Similar findings have been reported in the literature, where poor vertebral bone quality has been directly correlated with diminished pedicle screw stability [29–31]. Specifically, Kim et al. reported that Hounsfield Unit measurements below 100 at the L1–L4 levels markedly increased the risk of screw loosening [32]. Although DXA provides a global assessment of bone mineral density, it does not reflect localized pedicle bone quality. Recent studies have demonstrated that Hounsfield Unit measurements obtained from CT scans may offer superior predictive value for pedicle screw loosening by directly assessing trabecular bone density at the screw–bone interface. The absence of HU-based analysis represents a limitation of the present study and should be addressed in future prospective investigations.

Pain score analyses demonstrated that postoperative low back pain VAS scores was significantly higher in patients who developed screw loosening (*p* < 0.001). This finding suggests that screw loosening is not merely a radiological observation but also correlates with clinically significant symptoms. While previous studies have noted that some patients with screw loosening remain asymptomatic and do not require clinical intervention, the notably higher pain scores in our cohort highlight the potential clinical impact of this complication and raise the possibility of revision surgery being necessary in such cases [28, 33]. Those with postoperative back pain should be monitored more closely. The corset period can be extended, and work can be done to improve bone quality.

## Conclusion

In our study, semi-rigid stabilization achieved with PEEK rod systems was found to maintain screw loosening rates at an acceptable level while largely preserving overall spine stability. Nevertheless, it became evident that surgical technique errors and patient-specific risk factors could compromise the biomechanical advantages offered by these implants. In particular, careful preoperative assessment of bone quality, optimal screw placement, and the adoption of appropriate strategies for segment selection are critical for minimizing the risk of screw loosening. Based on these findings, it can be concluded that PEEK rod systems, when applied with meticulous patient selection and surgical execution, provide an effective and reliable means of spinal stabilization.
